# An estimation of the prevalence of diabetes mellitus and diabetic retinopathy in adults in Timor-Leste

**DOI:** 10.1186/s13104-015-1171-3

**Published:** 2015-06-18

**Authors:** Rosie Claire Hewitt Dawkins, Genevieve Frances Oliver, Manoj Sharma, Basilio Martins Pinto, Belmerio Jeronimo, Bernadete Pereira, Julia Magno, Lara Alexandra Motta, Nitin Verma, Mark Shephard

**Affiliations:** Sentru Matan Nasional, Hospital Nacional Guido Valadares, Bidau, Dili, Timor-Leste; Timor-Leste Eye Program, Royal Australasian College of Surgeons, Melbourne, Australia; Fred Hollows Foundation of New Zealand, Auckland, New Zealand; Flinders University International Centre for Point-of-Care Testing, Flinders University, Adelaide, Australia; Department of Ophthalmology, University of Tasmania, Hobart, Australia; School of Medicine, Sydney University, Sydney, Australia

**Keywords:** Timor-Leste, East Timor, Diabetes mellitus, Diabetic retinopathy, Point-of-care testing, HbA1c, Cataract

## Abstract

**Background:**

Once considered an affliction of people in high-income countries, diabetes mellitus is increasingly seen as a global epidemic. However, for many countries very little is known about the prevalence of diabetes and its complications. This study aims to estimate the prevalence of diabetes, and diabetic retinopathy, in adults in Timor-Leste.

**Methods:**

From March 2013 to May 2014, adult patients being assessed for cataract surgery at the Sentru Matan Nasional (National Eye Centre) in Dili, Timor-Leste had a point-of-care HbA1c measurement performed on the DCA Vantage device (Siemens Ltd) under a quality framework. A diagnostic cut-off of 6.5% (48 mmol/mol) HbA1c was used for diagnosis of diabetes. Ocular examination, blood pressure, demographic and general health data were also collected. Diabetic retinopathy assessment was carried out by ophthalmologists.

**Results:**

A total of 283 people [mean age 63.6 years (range 20–90 years)] were tested and examined during the study period. Forty-three people (15.2%) were found to have diabetes, with a mean HbA1c of 9.5% (77 mmol/mol). Of these, 27 (62.9%) were newly diagnosed, with a mean HbA1c of 9.7% (83 mmol/mol) and a range of 6.6–14% (49–130 mmol/mol). Nearly half (48.1%) of people newly diagnosed with diabetes had an HbA1c over 10.0% (86 mmol/mol). Of those with known diabetes, only 68.8% were receiving any treatment. Mean HbA1c for treated patients was 9.9% (85 mmol/mol). Diabetic retinopathy was identified in 18.6% of people with diabetes, of whom half had no previous diagnosis of diabetes.

**Conclusions:**

This study estimates the prevalence of diabetes at 15% in adults in Timor-Leste, a substantial proportion of whom have evidence of diabetic retinopathy. This is consistent with regional estimates. With the majority of patients undiagnosed, and management of people known to have diabetes largely inadequate, point-of-care testing is a valuable tool to assist with diabetes case detection and management. Whilst only a preliminary estimate, our data provides important impetus for further investigation of the prevalence and impact of diabetes in Timor-Leste. It provides guidance that further investment is required in expanding testing, as well as in prevention and treatment.

## Background

Diabetes is becoming one of the leading causes of morbidity and mortality worldwide. Once considered a disease of affluent countries, rates of diabetes are now increasing in low- and middle-income countries. It is estimated that worldwide around 346 million people suffer from diabetes, with 80% of these in low- and middle-income countries [1].

Interestingly, the pattern of development of diabetes in Asian countries is also more severe, with the disease typically appearing at a younger age, and generally with more devastating consequences [2]. Apart from the human toll, it is possible that hard-won economic gains could be undermined by morbidity in the working age population.

Timor-Leste is one of the world’s newest countries, having regained its independence in 1999 following a referendum. It was recognized by the United Nations in 2002. During the 24-year period of Indonesian rule there were between 102,800 and 183,000 excess civilian deaths [3]. After the vote for independence almost the entire health workforce left, and around 70% of all buildings were destroyed [4]. The country faces many challenges with 37.4% living below the international poverty line of US$1.25/day, and 50% literacy [5]. Hunger, malnutrition and child stunting are rife [6]. However, at the same time urbanization and dietary habits are changing rapidly. Although Timor-Leste remains a predominantly rural nation (70.4%), its rate of urbanization (4.18% per year) exceeds its growth rate (2.40% per year), largely driven by migration to Dili in search of cash employment [7].

Timor-Leste is currently in the process of building an entire health system from the ground up. At present health monitoring is weak, and resources for health are poor. Challenges continue in the prevention and treatment of communicable diseases, and non-communicable diseases such as diabetes.

Very little research has been undertaken to investigate the prevalence of diabetes and the impact of its sequelae in Timor-Leste. One pilot study in 2010 estimated prevalence to be approximately 5% in adults aged 40 years or over, with 74% of patients identified previously undiagnosed [8]. Anecdotally, doctors working in Timor-Leste, particularly in Dili, believe that the prevalence of diabetes is higher than previously reported. Furthermore, this seems low compared to Timor-Leste’s neighbouring countries. The International Diabetes Federation estimates prevalence in the Western Pacific region amongst adults aged 20–79 years as 8.6% [9]. Data from Indonesia is inconsistent but one study conducted in a population aged over 20 years in Ternate City, North Moluccas Province, Indonesia estimated prevalence at 19.6%, based on fasting glucose [10].

If left undiagnosed and untreated, diabetes can be a devastating disease. Microvascular end organ damage is the final common pathway, resulting in infections and amputations via neuropathy, renal failure via nephropathy, and blindness via retinopathy. This occurs via a number of pathways including activation of protein kinase C (PKC), increased expression of vascular endothelial growth factor (VEGF) [11] and advanced glycation end products (AGE) [12]. Glycation of proteins, lipids, nucleic acids and other molecules interfere with normal functioning. The retina lends itself particularly well to the monitoring of microvascular disease, as the vessels can be monitored by clinicians.

A temporal pattern of the emergence of diabetes mellitus and the development of diabetic retinopathy has been shown. Therefore, examination of patients for diabetic retinopathy gives some estimate of the length of time that they have had diabetes mellitus, and the severity of their disease. Although estimates vary widely, it is thought that almost all patients with Type I diabetes and more than 60% of patients with Type II diabetes will have retinopathy after a decade of disease [13]. Across published studies prevalence of retinopathy in known diabetes is 27.9%, and 10.5% in newly diagnosed diabetes [14].

An important step forward in the monitoring of diabetes was the global standardization of HbA1c testing methods, resulting in a strong push by international expert groups and professional organisations to use the HbA1c tests for the diagnosis of diabetes [15, 16]. In 2010 the American Diabetes Association released new guidelines stating that diabetes need not be diagnosed by the gold standard oral glucose tolerance test, but could instead be diagnosed using glycated haemoglobin, with an HbA1C of ≥6.5% (48 mmol/mol) as the cut off point for the diagnosis of diabetes [17].

Considerable work has been conducted across many parts of the world, primarily using receiver-operator-curves to compare the oral glucose tolerance test with HbA1c, to create local diagnostic cut-off points for the diagnosis of diabetes. All of these revised estimates have been around or lower than 6.5% (48 mmol/mol). This indicates that using a diagnostic cut off of 6.5% (48 mmol/mol) should provide a good, if conservative, estimate of the proportion of patients with diabetes [18].

Furthermore, the development of rapid point-of-care HbA1c tests has made opportunistic testing for diabetes mellitus much easier. This is of considerable importance in a country such as Timor-Leste where the health system is weak, laboratory access is limited, and patients may have very few interactions with the health system. Indeed, there is no HbA1c test available at the Hospital Nacional Guido Valadares, other than at the eye clinic. Through a partnership with the Flinders University International Centre for Point-of-Care Testing (iPOCT), the National Eye Centre in Dili was able to conduct point-of-care testing for HbA1c using the DCA Vantage (Siemens Ltd) as part of an international model developed by iPOCT called the ACE (Analytical and Clinical Excellence) Program. This device has been shown to produce point-of-care test results that are analytically equivalent to laboratory measurements of HbA1c [19–21].

In addition to diabetes, another globally important non-communicable disease is arterial hypertension. The American Heart Association defines hypertension as a blood pressure of greater than 140/90 [22]. Hypertension is associated with an increased risk of heart disease and stroke. Furthermore, hypertension is a potent synergistic factor in the worsening of diabetic retinopathy [23].

Cataract is an important cause of preventable blindness across the world, especially in low-income countries [24]. Formation of cataract is multi-factorial, with the most important risk factor by far being advancing age, though also including diabetes and smoking [25]. In Timor-Leste cataract is the leading cause of blindness [26]. Furthermore, the eye care system is one of the most well developed parts of the health care system in Timor-Leste with a permanent National Eye Centre in Dili and outreach to all of the districts. Therefore, the population of patients presenting to the National Eye Centre for workup for cataract presents a very useful opportunistic population in which to study the prevalence of diabetes.

## Methods

In this study health professional staff from the National Eye Centre performed point-of-care HbA1c testing on all patients being assessed for cataract surgery at the Centre in Dili, Timor-Leste. Staff undertook a training and competency assessment program and performed regular quality control testing (as part of their participation in the Analytical and Clinical Excellence [ACE] Program) to monitor analytical performance of the DCA Vantage device. We used a level of ≥6.5% (48 mmol/mol) as diagnostic of diabetes. Additionally, we included patients diagnosed and treated for diabetes mellitus, but who now had an HbA1c of <6.5% (48 mmol/mol). Patients over the age of 18 were eligible for inclusion in this study.

Demographic data including age and sex were collected. Data regarding reported family history and medication history was also collected from patients identified with diabetes.

Clinical examination was performed including visual acuity, intraocular pressure, anterior and posterior segment examination, and sphygmomanometry. When the retina could not be examined, patients were asked to present again for examination after cataract surgery. When diabetic retinopathy was found it was graded by an ophthalmologist according to the International Clinical Disease Severity Scale for Diabetic Retinopathy [27].

Patients who required examination further to standard care for cataract surgery provided written informed consent. Consent forms were approved in the human research ethics approval process, and were available in Tetun and English. Data was de-identified and securely stored.

Patients identified with diabetes, hypertension, or the end organ consequences of these diseases were referred to the internal medicine clinic at Guido Valadares National Hospital, or to a local doctor.

Ethics approval for this study was received from the Timor-Leste Ministry of Health, and the tenets of the Declaration of Helsinki were abided by at all times.

## Results

283 patients were included in the study over a 14-month period between March 2013 and May 2014. This represents a consecutive series of adult cataract patients, with exceptions for periods of time when the HbA1c device was unavailable for testing (due mainly to delays in delivering testing reagents).

Slightly more than half (58.7%) of patients were male. The age range was 20–90 years, with a median of 66 years, and a mean of 63.6 years. Male patients had a slightly greater age range (20–90 years) than female patients (31–86 years) (Table 1).

**Table 1:** Demographic Data

	Female	Male
Proportion of total 283 subjects (%)	41.3	58.7
Mean age (years)	63.6	
Median age (years)	66	
Age range (years)	20–90	

Reported HbA1c results ranged from 4.2 to 14% (22–130 mmol/mol) (with 14% being the upper limit of the device’s measurement range) (Table 2). For the recruited population, the mean HbA1c was 6.1% (43 mmol/mol). For patients without diabetes the mean HbA1c was 5.5% (37 mmol/mol).

**Table 2:** Diabetes and diabetic retinopathy in the adult cataract surgery population in Dili, Timor-Leste

	Units	All patients	Patients without diabetes	Patients with diabetes HbA1c ≥6.5% (48 mmol/mol)		
				All	Diagnosed during study	Previously diagnosed with diabetes
Number of subjects		283	240	43	27	16
Mean HbA1c (95% CI)	%	6.1 (5.9–6.3)	5.5 (5.45–5.55)	9.5 (8.8–10.2)	9.7 (8.9–10.6)	9.2 (8.2–10.2)
	mmol/mol	43 (41–45)	37 (36–37)	80 (73–88)	83 (74–92)	77 (66–88)
HbA1c range	%	4.2–14	4.2–6.4	6.5–14	6.6–14	6.2–12.9
	mmol/mol	22–130	22–46	48–130	49–130	44–117
Percentage of subjects with diabetic retinopathy		–	–	18.6% (n = 8)	14.8% (n = 4)	25.0% (n = 4)

Forty-three patients (15.2%) were found to have diabetes, with a mean HbA1c of 9.5% (80 mmol/mol). Of these, 27 (62.9%) were newly diagnosed, with a mean HbA1c of 9.7% (83 mmol/mol) and a range of values from 6.6 to 14% (49–130 mmol/mol). Thirteen of the newly diagnosed patients with diabetes (48.1%) had an HbA1c over 10.0% (86 mmol/mol).

Amongst the 16 patients with known diabetes, the duration of diagnosis ranged from 2 months to 13 years, with a mean duration of 4.9 years. Eleven patients (68.8%) were on treatment, either medical or lifestyle. Mean HbA1c for treated patients was 9.9% (85 mmol/mol). Three treated patients had achieved HbA1c <7.0% (53 mmol/mol).

The point-of-care device’s performance was analytically sound, with 100% of quality control test results for HbA1c being within the goal for allowable limits for acceptable analytical performance recommended for laboratories by the Royal College of Pathologists of Australasia’s Quality Assurance Program for Glycohaemoglobin.

Diabetic retinopathy was identified in eight of the 43 patients with diabetes (18.6%), including four with previously undiagnosed diabetes. No proliferative retinopathy was found, though severe non-proliferative retinopathy was found in one patient who was previously undiagnosed.

Blood pressure was measured and recorded for 245 patients (86.6%). The range of systolic blood pressure (SBP) was 74–220 mmHg, with a mean of 136 mmHg. 114 patients (46.5%) had SBP ≥140 mmHg, systolic hypertension. Amongst those patients with diabetes, the SBP range was 90–195 mmHg, with a mean of 143 mmHg (values were available for 83.3% of diabetic patients).

## Discussion

This study shows the great potential burden of diabetes amongst adults in Timor-Leste. The prevalence in our population of unselected adult cataract patients was 15.2%, much higher than a previous estimate, but similar to regional estimates.

Furthermore, we show that the vast bulk of people with diabetes are undiagnosed (62.8%), and those that are diagnosed are inadequately managed—either untreated or inadequately treated. This is likely due to poor access to diagnostic testing and monitoring, and unreliable medication supply. The issues around management of diabetes are clearly highlighted by the finding that the group of treated patients had higher mean HbA1c than those that were newly diagnosed at the National Eye Centre.

Point-of-care testing has provided a clinically useful and analytically sound tool to assist the diagnosis of diabetes in a country where HbA1c is not available through any hospital laboratory services in Timor-Leste. Ongoing access should mean that physicians can better assist patients to manage levels of glycaemia, provided access to medication also improves.

In this study the rates of diabetic retinopathy in known (25.0%) and previously undiagnosed (14.8%) patients with diabetes are comparable to reported values for these groups in the literature [14]. We therefore conclude that other microvascular complications of diabetes such as neuropathy, nephropathy, cardiac and central nervous system complications are likely to be at similar to literature rates. Whilst this requires significant further study, it is of significant consequence to the health care system in Timor-Leste.

Although the study was not designed to report on systemic hypertension, the proportion of patients with hypertension in this population (46.5% with SBP ≥140 mmHg) is cause for concern in terms of likely consequences for morbidity and mortality.

An important limitation of this study is that it was undertaken in a specific population, a population of cataract surgery patients. However, given that cataract is a relatively ubiquitous disease in older age, and because diabetes is a disease of significant importance, we believe that our study population is a reasonable population in which to make an initial estimate of the prevalence of diabetes in adults in Timor-Leste. It is important to bear in mind the relationship between cataract and diabetes, and the increasing prevalence of diabetes with advancing age. Therefore, the prevalence of diabetes may be higher in our study population than the general population, and additional work should be undertaken in a broader population-based setting.

Another limitation of this study is that it was conducted in Dili, the capital city of Timor-Leste which is relatively more urbanised and less poor than rural districts. This makes it likely that rates of diabetes may be higher than in other parts of the country. However, internal migration to Dili, and other regional centres is proceeding at a rapid rate, as is economic development in Timor-Leste.

## Conclusions

This study shows that, despite its status as a new and relatively economically undeveloped country, the prevalence of diabetes in Timor-Leste is very likely at least similar to countries in the region. This is important because the prevalence has previously been unknown but thought to be low, and it has not been a focus for the health system.

Our data shows that diabetes, and likely other non-communicable diseases such as systemic hypertension, are becoming an important source of morbidity and likely mortality in Timor-Leste as in other developing countries. Further epidemiological research is required to more accurately estimate the prevalence and burden of diabetes in Timor-Leste, but from our data it seems that left unchecked the humanitarian and economic burden will be very large in a fragile health system and economy.

This study shows that it is urgent that significant investment is made by the Government of Timor-Leste, as well as external donors, in health system strengthening to meet these emerging complex needs. Building greater on-ground capacity to undertake quality-assured point-of-care testing is one step to assist this investment. Capacity building around prevention and treatment are also required.

## Abbreviation

HbA1c: glycated haemoglobin
